# Predicting the Impact of Transforming the Medicaid Program on Health Centers’ Revenues and Capacity to Serve Medically Underserved Communities

**DOI:** 10.1111/1468-0009.12426

**Published:** 2019-10-16

**Authors:** ANNE ROSSIER MARKUS, KAN GIANATTASIO, ERIC (QIAN) LUO, JULIA STRASSER

**Affiliations:** ^1^ Milken Institute School of Public Health The George Washington University

**Keywords:** Medicaid, block grants, Affordable Care Act, repeal and replace, community health centers

## Abstract

**Policy Points**

- Recent federal proposals to use block grants or per capita caps to fund Medicaid would likely lead to cuts in Medicaid funding for health centers, which are an important source of care for Medicaid enrollees.
- Recent Medicaid §1115 waivers are seeking to change state‐level enrollment and eligibility requirements in ways that are expected to adversely affect health center revenues.
- Proposed Medicaid funding cuts are expected to lead to reductions in service capacity across all health centers over the long term.
- State policymakers should understand the likely impacts of proposed Medicaid program changes on health centers in their states and allocate funding to help offset lost federal financing.

**Context:**

In 2017, Congress considered implementing block grants or per capita caps to significantly reduce federal financing of the Medicaid program. Medicaid plays a key role in supporting health centers in their provision of care to patients with Medicaid coverage. Consequently, changes to the program could have serious implications for health centers and their ability to fulfill their mission.

**Methods:**

We used a mixed‐methods approach to (a) test a model simulating the effect of block grants and per capita caps on health centers’ total revenues and general service capacity, and (b) augment model assumptions by using information collected from official Medicaid documents and interviews with health center leadership staff. Data came from the Uniform Data Systems (UDS), state‐ and county‐level population projections, structured analyses of waiver documents, and interviews with health center leaders in seven states with approved or pending Medicaid §1115 waivers.

**Findings:**

By 2024, in states where Medicaid coverage was expanded under the Affordable Care Act, block grant funding for Medicaid would decrease total health center revenues for the expansion population by 92%, and by 58% for traditional enrollees. In nonexpansion states, block grants would decrease health center revenues for traditional Medicaid enrollees by 38%.

In expansion states, a per capita cap would, by 2024, decrease health center revenues for the expansion population by 78%, and for traditional Medicaid enrollees by 3%. The per capita cap would reduce health center revenues for traditional Medicaid enrollees in nonexpansion states by 2%.

Eliminating the Medicaid expansion population would not fully compensate for health center revenue deficits in expansion states. Health center executives in all sample states expressed significant uncertainty around federal plans to reduce Medicaid funding as well as the financial implications of §1115 waiver requirements. Many interviewees anticipate cutting back on services and/or staff as a result.

**Conclusions:**

Both block grants and per capita caps would have a detrimental effect on health centers. Although health center leaders anticipate a reduction in services and/or staff, the uncertainty around federal and state proposals hinders health centers from making concrete strategic plans. States should prioritize communicating changes to health centers in a timely manner and be prepared to set aside dedicated funding to address anticipated shortfalls.

In 2017, congress considered changes that would have significantly reduced federal financing of the Medicaid program. These changes involved several options, including capping program funding through a per capita cap or lump‐sum (ie, block grant) mechanism. If Congress were to fund Medicaid through block grants or limit federal financial liability in some other way, states would need to make difficult choices regarding Medicaid program eligibility, scope of benefits, and/or provider payments to maintain the program's financial sustainability while balancing state budgets.

Medicaid plays a key role in supporting health centers in the provision of care to patients who have Medicaid coverage. Nationally, Medicaid patients make up almost half of all health center patients, and Medicaid revenues are typically the largest source of funding for health centers.^1^ Consequently, changes to the Medicaid program could potentially have serious implications for health centers and their ability to fulfill their mission.

This study uses a mixed‐methods approach to achieve the following two aims:

- To test a quantitative model that simulates the effect of Medicaid per capita caps and lump‐sum block grants on total health center revenues and general capacity to serve medically underserved communities, and
- To directly inform and refine model assumptions with structured qualitative analysis of official state and federal Medicaid documents and interviews with health center leaders in states with approved or pending Medicaid §1115 waivers.

The qualitative analysis serves two purposes. First, it is important to understand current state‐level Medicaid design decisions and whether they are a likely indicator of the direction a state might take if given the opportunity to use block grant funding for the program, especially since federal proposals remain vague on a number of important points, such as how reimbursement models might change for health centers. Second, systematically collected qualitative data can confirm or augment underlying theoretical assumptions in quantitative simulation efforts, thereby helping us assess our model and its implications for public policymaking.

This article presents the results of the simulation model and the information gleaned from interviews. Specifically, we compare the likely effect of Medicaid lump‐sum block grants and per capita caps on total health center revenues and, consequently, the ability of health centers to retain medical personnel to maintain the level of service the centers currently provide. We made a number of assumptions in building the model; these are derived in part from federal bills and the provisions affecting only Medicaid, and in part from our understanding of what health center leaders told us about how each scenario would play out in their particular state and health center.

This article also places the study and its results within the broader context and recent history of the Medicaid program, starting in 2010 when the Patient Protection and Affordable Care Act (ACA) was enacted with the intention of creating a minimum national floor of health care coverage for all low‐income individuals regardless of place of residence. We end with a discussion of our findings and their implications for health centers’ ability to maintain their mission if the nature of the Medicaid program changes in the foreseeable future. Efforts to fund the Medicaid program through block grants tend to resurface on a regular basis, and the results presented in this article will enable states and health center leaders to position themselves in future debates with pertinent data that are not typically available at the provider level with this level of specificity.

## Medicaid as a National Minimum Floor of Coverage

The Medicaid program is the public health insurance safety net of the US healthcare system, providing insurance coverage to over 75 million of the country's most vulnerable individuals across the age spectrum.^2^ It is funded and administered jointly by the federal government and state governments. Currently, for every dollar that a state spends on the program, the federal government provides at least equal funding.^3^ The rate at which the federal government matches state funding is determined by the Federal Medical Assistance Percentage (FMAP) and is inversely proportional to state per capita income; therefore, federal funding has redistributional effects across states. The wealthiest states have an FMAP of 50% (the statutory minimum), resulting in a dollar‐to‐dollar match; the poorest state in 2018—Mississippi—had an FMAP of just under 76% (although the statutory maximum was 83%) and received $3.11 in federal funding for every $1 it spent.^3^, ^4^ Under this federal‐state partnership, states enjoy significant leeway in many design and implementation features of the Medicaid program, as long as those features fall within the general requirements set forth by federal law.

Prior to the passage of the ACA, Medicaid coverage was mandated for four groups of low‐income individuals: children, parents of dependent children, pregnant women, and Supplemental Security Income (SSI) recipients (ie, the elderly, blind, and disabled). The income eligibility thresholds for traditional Medicaid coverage varied from group to group and from state to state.^5^ Eligibility was (and remains) most generous for children and pregnant women. As of January 1, 2019, state upper eligibility levels ranged from 133/138% to 405% for children and 133/138% to 322% for pregnant women.^5^ In 2013, the median eligibility threshold was 64% of FPL for parents of dependent children, and the median threshold was capped at 100% of FPL for SSI recipients across states.^6^

Until the ACA expanded Medicaid, childless, nonpregnant, and nondisabled adults did not qualify for Medicaid in most states.^6^ The expansion was intended to extend coverage on a national level to *all* adults whose family income was less than 138% of FPL (133% with a 5% income disregard). However, 26 states (led by Florida) issued a legal challenge to the expansion on the grounds that it was constitutionally coercive. The US Supreme Court ultimately ruled five to four in favor of the plaintiffs on June 28, 2012, making expansion optional for states and underscoring how Medicaid had “transformed [from a program targeting a limited number of low‐income groups] into a program to meet the health care needs of the entire nonelderly population with income below 133 percent of the poverty level.”^7^

Unlike traditional Medicaid, the federal government funded 100% of the costs of covering the expansion group until 2016; after 2016, the federal match rate began to decrease; it is slated to reach 90% by 2020.^8^ Twenty‐four states and the District of Columbia opted for Medicaid expansion to go into effect by no later than January 1, 2014. Seven additional states opted for expansion by mid‐2016, and Maine voters and Virginia lawmakers approved Medicaid expansion in November 2017 and May 2018, respectively.^9^ Most recently, voters in Idaho, Nebraska, and Utah voted for Medicaid expansion in their states in 2018 ballot initiatives, but voters in Montana rejected a ballot initiative to repeal the sunset of the Medicaid expansion provision in June 2019. In 2016, more than 15 million adults were enrolled in expanded Medicaid programs across the 32 expansion states.^10^

## Medicaid as a Time‐Limited, Transitional Program Toward Self‐Sufficiency

In 2017, both the US House and Senate proposed a series of bills and amendments to dismantle the ACA and fundamentally change the way in which the federal government funds the Medicaid program. The House passed the American Health Care Act (AHCA) in May 2017. In the Senate, the Better Care Reconciliation Act (BCRA) was voted out of the Budget Committee in June 2017 and the Graham‐Cassidy‐Heller‐Johnson amendment (GCHJ) was proposed in September 2017; however, neither of these measures passed the Senate, so the House and Senate bills could not be reconciled and sent to the president for signing.

If these legislative efforts had succeeded, Medicaid financing would have been converted to a per capita cap, ending the policy of open‐ended funding that has existed since Medicaid's inception and changing coverage for the Medicaid expansion population.^11^, ^12^, ^13^, ^14^, ^15^, ^16^ Starting in 2020, all three proposals would have generally limited the growth in federal per capita Medicaid payments to the states to the medical care component of the consumer price index (CPI‐M) for children and nondisabled adults, and to CPI‐M + 1 percentage point for SSI recipients.^11^, ^12^, ^13^, ^14^, ^15^

If AHCA had become law, this scheme would have continued after 2024. Under both BCRA and GCHJ, the per capita caps would have become even more stringent starting in 2025. The BCRA's limits on per capita growth in federal Medicaid funding for all enrollee groups would have been determined by the urban consumer price index (CPI‐U), which grows slower than CPI‐M. Under the GCHJ, per capita funding for children and adults would have been adjusted by CPI‐U and per capita funding for SSI recipients would have been adjusted by CPI‐M. Additionally, each of the three proposals also would have allowed states to opt for a block grant to cover children and/or nonexpansion, nondisabled, and nonelderly adults in lieu of the per capita cap mechanism. The block grant amounts would have been equivalent to the federal portion of each state's target per capita expenditures in the base year (as determined by the state FMAP), multiplied by the number of enrollees in the base year; total amounts would be adjusted annually to account for population growth and inflation (based on the CPI‐U).^11^, ^13^, ^15^

These new mechanisms would have significantly reduced federal contributions to the Medicaid program compared to the status quo.^12^, ^14^ According to the Congressional Budget Office (CBO) calculations, federal outlays to Medicaid between 2017 and 2026 would have been reduced by $834 billion under AHCA and by $738 billion under BCRA.^12^, ^14^ In addition, the three proposals offered states the option to add work requirements as a condition of Medicaid eligibility (and as a means to control spending).^11^, ^13^, ^15^

## Section 1115 Medicaid Demonstration Waivers and State Medicaid Changes

The Medicaid §1115 demonstration waiver authority was established to offer states the opportunity to waive certain federal requirements so they could experiment with budget‐neutral ways of implementing the Medicaid program more efficiently in ways that promote program goals of providing access to medical assistance.^17^, ^18^ Over the years, waivers have been used widely and variably to promote the priorities set forth by the Centers for Medicare and Medicaid Services (CMS), most notably to expand coverage and/or services or to achieve cost‐savings through managed care plans.^18^

After the ACA became law, a handful of states invoked the §1115 waiver authority to implement alternative ACA Medicaid expansion models that employ design features outside of the scope authorized by the ACA.^19^ For example, states have used the waiver authority to implement work requirements as well as features that mimic commercial insurance plans and/or increase the burdens on individuals seeking to gain and maintain Medicaid coverage, such as health savings accounts and rewards plans; healthy behavior incentives; premiums and cost‐sharing requirements; and disenrollment with or without eligibility lockouts for failure to pay premiums or failure to meet eligibility renewal requirements. Notably, these changes, together with a block grant and/or per capita cap, could compound the loss of Medicaid coverage for health center patients and thus revenues for health centers.

Coinciding with the 2017 Congressional legislative proposals, administrators from CMS and the US Department of Health and Human Services (HHS) issued guidance encouraging new approaches to §1115 waivers. In a letter penned in March 2017, HHS Secretary Thomas Price and CMS Administrator Seema Verma jointly urged state governors to leverage the waiver authority to incorporate work/community‐engagement requirements as a condition of eligibility into the Medicaid program, as well as design features mimicking commercial insurance plans, including the inability to invoke retroactive eligbility.^20^ The call for work requirements was reasserted by Verma in a speech to the National Association of Medicaid Directors in the fall of 2017, which also highlighted aims to “turn the page” in the Medicaid program and move away from a “cookie cutter” Medicaid design by increasing state flexibility.^21^ CMS demonstrated its commitments to these goals by publishing new policy guidelines supporting states in these efforts in January 2018.^22^

## Medicaid and Federalism

The recent changes that have been proposed and implemented at the federal and state levels signify a fundamental shift in the spirit of the Medicaid program. These changes, however, are not entirely new. Block grant funding for Medicaid was first proposed almost four decades ago by President Ronald Reagan in 1981, and the proposal was later revived by Speaker of the House Newt Gingrich in 1995 and President George W. Bush in 2003, although none of these proposals were ever implemented.^23^ As Lambrew writes, block grant funding for Medicaid has been popular among Republicans because it (a) promotes federalism by granting states more flexibility and control over program design, and (b) controls spending on entitlement programs and eliminates unpredictability in the budget, and is thus more fiscally conservative.^23^

The 2017 federal proposals for per capita caps with a block grant option would have met these goals. The per capita cap option could help shield states against events that lead to greater demand for Medicaid (eg, economic downturns), but the caps would limit states’ abilities to expand the scope of service coverage to include new (and expensive) services, increase provider payment rates, or invest in capacity. Conversely, block grants would allow states more flexibility in enhancing the scope of benefits, but they would not offer protection against increased demand for Medicaid coverage in the event of an economic downturn, catastrophic events, or other unforeseen increases in Medicaid utilization. Under either the per capita cap or block grant option, states would need to make difficult choices regarding Medicaid program eligibility, the scope of benefits, and/or provider payment to ensure the financial sustainability of the program, and these choices would inevitably affect health centers’ abilities to further their mission.

## Health Center Operations Under the Current Medicaid Program

**Figure 1:**
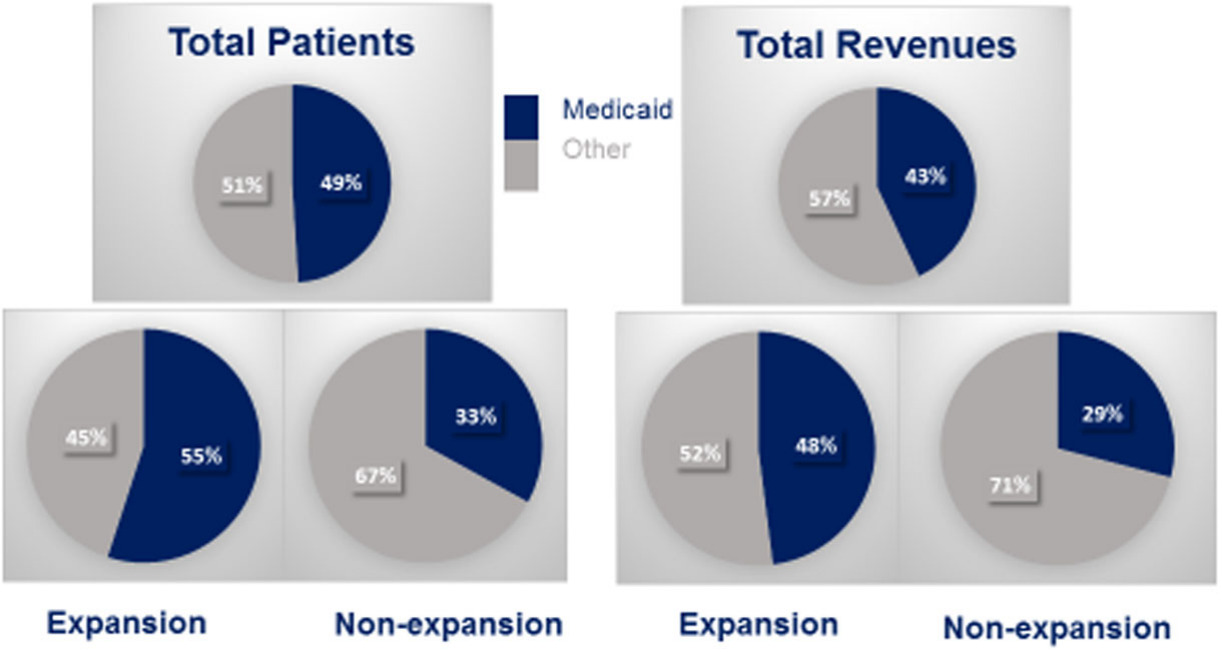
Medicaid as a Source of Health Center Patient Coverage and Revenue by State Medicaid Expansion Status, 2016 ^a^“Other” includes revenues from Medicare, private insurance, self‐paying/uninsured patients, Section 330 grants, other grants and contracts, and miscellaneous sources. Data from Rosenbaum, Tolbert, Sharac *et al*., 2018.^1^ [Color figure can be viewed at http://wileyonlinelibrary.com]

As discussed previously, Medicaid patients make up almost half of all health center patients, and Medicaid revenues are typically the largest source of funding for health centers (Figure 1).^1^ In 2016, 12.7 million of the 25.9 million patients served by health centers were Medicaid patients, and national health center revenues from Medicaid were approximately $10.2 billion, about 43% of the total national health center revenues of $23.8 billion.^1^

As providers whose services are a mandatory benefit under Medicaid, health centers are reimbursed differently from other providers that participate in the Medicaid program. Since 2000, federal law has required that state Medicaid agencies implement a prospective payment system (PPS) or an alternative payment method (APM) to determine all‐encompassing rates for health center encounters or visits, which are adjusted annually using the Medicare Economic Index (MEI) and when the scope of services included in the rate changes (eg, to reflect an increase in service capacity).^24^

States may determine which services are included in the rate and can impose limits on how many encounters can be billed per member per day. Additionally, when health centers contract with Medicaid managed care organizations, they must receive payment that is equal to the PPS rate. When managed care rates are lower than the PPS rate, health centers are eligible to receive a supplemental payment (known as the “wrap” or “wraparound”) to make up the difference. Although states already have some flexibility to design their payment policy for health centers, some states have advocated for more flexibility under proposed and implemented §1115 waivers; in contrast, health center leaders argue that payment policies must include protections to ensure that reimbursement reflects the cost of treating Medicaid patients.^25^

Although the federal block grant/per capita cap proposals did not specifically address how health centers might be paid and reimbursed, anecdotal evidence from experts suggests that the wraparound rate would likely be eliminated if that were permitted under federal regulations. The interviews presented in this article did not cover what would happen to the PPS/APM system in place in a given state, as even the broader issue of how a block grant or a per capita cap might be designed remained vague, with specific features nonexistent or unavailable at the time of the interviews.

Medicaid funding has been a key factor in building, maintaining, and boosting the capacity of health centers to provide needed services to their local communities. In addition, the federal health center grant funding that supports much of the care delivered to uninsured patients at health centers (known as Section 330 grants) has also been credited as an important source of revenue that can support capacity building. Also, under the ACA, additional revenues have flowed to health centers from patients’ improved insurance coverage, particularly through Medicaid expansion in expansion states, and from growing federal grant funding through the Community Health Center Fund.^26^ Previous interventions, such as the implementation of electronic health records and the building of new clinics as additional access points for health center patients, have also contributed to the growth in health center capacities. Factors that have challenged capacity building have included the difficulty in recruiting and retaining clinicians, operating in communities lacking resources, insufficient grant funding, and inadequate physical space.^26^ By targeting Medicaid federal funding, federal proposals to use block grants constitute a particular threat to health centers’ ability to meet the demand for their services from Medicaid and other patients.

## Research on the Effects of Medicaid Expansion, Work Requirements, and Premium/Cost‐Sharing Initiatives

A growing body of empirical research supports the notion that the ACA has substantially reduced the uninsured rate and changed the provider‐payer mix by providing public or private insurance to previously uninsured individuals (including those who were eligible but uninsured).^27^, ^28^ Studies focusing on the impact of the Medicaid expansion have capitalized on the natural experiment resulting from the US Supreme Court decision to make the expansion optional for states by comparing the experiences of Medicaid expansion states to those of nonexpansion states. Studies of the impact of expansion on providers have almost exclusively focused on hospitals and their various facets.^27^ Evaluations of the ACA's impact on health centers are limited, but available analyses consistently show that health centers and their patients have benefited from expanded coverage, particularly in Medicaid expansion states.^29^, ^30^, ^31^ With the influx of new patients (most of whom are tied to Medicaid funding), health centers have experienced growth in their capacity to offer services and meet the demands of newly insured patients.^32^, ^33^

Although studies predating the ACA suggest that policies to reduce coverage have a disproportionately detrimental effect on lower income individuals, studies undertaken in the wake of the implementation of the ACA coverage expansions suggest that effects of coverage contractions will not necessarily mirror the effects of coverage expansions.^28^ It is unclear how health centers would fare as a network of providers (nationwide or in any given state) under projected reductions in coverage. Health centers would likely see a decrease in the portion Medicaid‐covered patients and a rise in the portion of uninsured patients, and by extension, a decrease in total Medicaid revenues. However, no study has yet documented this empirically. New Medicaid attributes being imposed through §1115 waivers—including work requirements, financial requirements, reporting requirements, healthy behavior incentives, and plans that resemble health savings accounts, are all consistent with the stated conservative principles of individual responsibility, self‐sufficiency, and self‐reliance.^2^, ^34^, ^35^ While no evidence of the impact of work requirements and other features on reaching these goals in health care exists, work requirements in particular echo the Temporary Assistance for Needy Families (TANF) welfare‐to‐work program authorized under the Personal Responsibility and Work Opportunity Reconciliation Act of 1996.^36^ Notably, the reach of Medicaid work requirements could far exceed that of TANF. In 2016, the broad work requirements of TANF covered 640,000 adults; in contrast, 22 million Medicaid enrollees (about 30% of total Medicaid enrollment) could be subject to work requirements nationwide.^37^ Although many of the Medicaid work requirements are too recent for a robust evaluation of their impact, preliminary evidence suggests that they may result in high rates of Medicaid disenrollment—up to 50% of enrollees subject to work requirements could be at risk of losing coverage.^37^ In Arkansas, when 10% of Medicaid expansion enrollees were subjected to the work requirements, anecdotal evidence suggests that approximately one‐quarter of them (about 7,000 people) were not able to comply with the reporting requirements.^38^

Premiums and cost sharing have been built into the Medicaid program in the past within federal limits or as an experiment though the waiver authority. As a permissible design feature, cost sharing is particularly common in the Children's Health Insurance Program (CHIP).^39^ The stated goals of these requirements are to promote personal responsibility; align CHIP coverage with the private insurance market, particularly as CHIP eligibility expanded to relatively higher income groups; and control state spending.^40^, ^41^ However, research has shown that introducing new premiums or increasing existing premiums in Medicaid/CHIP programs results in substantial disenrollment rates almost immediately (11% to 50%, depending on the size of the premium and the length of time postimplementation), with many of the newly disenrolled (20% to 70% across studies) citing cost as the primary reason for losing coverage.^42^

Other research has found that monthly caseloads among populations affected by Medicaid premiums decreased by 1% to 5% over the six months after existing premiums were raised (Kansas, New Hampshire), and by more than 18% after new premiums were imposed (Kentucky).^43^ Furthermore, there is evidence that copays result in higher unmet medical needs and greater financial pressures for Medicaid enrollees. In a study published in 2005, up to 35% of individuals eligible for Medicaid who were surveyed in Oregon reported that they did not obtain care due to cost, and 24% of individuals reported that they did not have the money to make the copayments, ultimately leading to increased appointment no shows.^44^ Similarly, in Utah, copays were associated with lower health service and prescription drug utilization, with over 40% of Medicaid beneficiaries surveyed reporting that even small, seemingly nominal copayments caused “huge problems” and “serious financial difficulties.”^42^, ^44^

It is essential to understand the potential impact of these and other requirements in recently approved and pending §1115 waivers granted to states. They may be a good indication of the direction that states would take should the federal government pass block grants and/or per capita caps for Medicaid funding.

## Methods

### Research Objectives and Questions

Given the crucial role of health centers as safety nets and Medicaid providers in many medically underserved and rural communities, it is important to better understand how proposed federal changes to the Medicaid program may affect their total revenues and their capacity to provide services. Specifically, our core aims/objectives and questions are as follows:

1. To test models based on Medicaid provisions of AHCA, BCRA, and GCHJ that simulate the effects of Medicaid block grants or per capita caps on health centers’ total revenues and capacity to serve medically underserved communities.
  a. What would be the impact of Medicaid block grants or per capita caps on total health center revenues in the aggregate, by location in Medicaid expansion versus nonexpansion states, and by state?
  b. What would be the impact of Medicaid block grants or per capita caps on health center staff capacity in the aggregate, by location in Medicaid expansion versus nonexpansion states, and by state?
2. To augment the model assumptions with information collected from official state and federal documents and interviews with health center executives (CEOs/CFOs) in states with approved or pending Medicaid §1115 waivers with work requirements and other coverage restrictions.
  a. What are the implications of the changes being considered by states with approved and pending §1115 waivers, and are they an indication of how states may design a block grant in the future?
  b. How are health centers in these states preparing for anticipated decreases in revenue and service capacity that would result from the proposed or to be implemented changes?

### Study Approach

We used mixed methods to approach this research. For the quantitative component of this study, we simulated forecasting models using data from the Uniform Data System (UDS), and for the qualitative component, we conducted systematic analyses of state and federal waiver documents, as well as semistructured, in‐depth interviews with health center leaders from seven states with implemented/pending §1115 expansion waivers. The statistical analyses were performed in Stata 15, and qualitative data management and analysis were performed in NVivo 11. This study was reviewed by the George Washington University Institutional Review Board and determined to be exempt research (IRB #121712).

### Quantitative Analysis

#### Data

Our main data source was the UDS data, which are maintained by the Health Resources and Services Administration (HRSA) Bureau of Primary Health Care and cover information collected from all health centers receiving federal Section 330 grants on an annual basis.^45^ UDS is a comprehensive dataset containing data on staffing, services provided, revenues, utilization, and geography, as well as select quality/outcome measures. We identified the universe of health centers in each state from years 2000 to 2016 for our analyses. The universe of health centers grew from 703 HCs in 2000 to 1,337 in 2016. Because of this rapid growth, our state‐level modeling approach incorporates health center service capacity growth in a given state. Additionally, we used Medicaid eligibility data for children, parents, and childless adults compiled by the Henry J. Kaiser Family Foundation.^6^ Finally, we drew annual historical state‐level population data from the US Census Bureau^46^ and state‐level population projections from the Weldon Cooper Center for Public Service at the University of Virginia.^47^

*Simulation Models, Assumptions, and Limitations*. We constructed a baseline model and three additional models to simulate the effects of block grant funding or implementing per capita caps in Medicaid, as proposed in all federal bills from 2017. (Note: We did not aim to score each bill for its specific Medicaid provisions; rather, we selected common parameters across the bills, such as proposed adjustments for inflation, to include as assumptions in our models.)

We limited our simulations to changes that would affect the Medicaid program. Therefore, our results do not account for some of the interactions that would be expected to happen between the Medicaid provisions and other provisions affecting health center revenue mix (eg, changes in the Community Health Center Fund). We also limited our simulations to reductions in the federal portion of Medicaid financing to states, even though states would likely make changes in their own financing of the program if faced with per capita caps or the choice to use block grant funding. In other words, we assumed that revenue shortages under each reform scenario were the result of federal contribution reductions relative to the baseline scenario only, and we held state behavior constant to 2016 levels, the baseline year. Similarly, because it is unclear whether and how Section 330 funding levels would respond to rollbacks in Medicaid eligibility due to block grants or per capita caps, we did not build any assumptions on changes to Section 330 funding in our models. Also, while we recognize that other potential legislative changes (eg, reducing subsidies for Health Insurance Marketplace plans or repealing the ACA's individual or employer mandates) would also likely affect overall health center revenues, those types of changes are outside the scope of our research questions and not modeled in our simulations. Furthermore, we note that health center behavior and patient demand would also be expected to be affected by the proposed changes because more people would be uninsured, but we were not able to model these assumptions in the approach we took. We stress that these various modeling limitations and potential dynamics should be considered in interpreting our simulation results. We simulated the results over the midterm (ie, the five year period 2020‐2024), during which we assumed no changes in the economy and no unforeseen events, such as catastrophic economic or natural disasters, that would affect healthcare demands.

Finally, for each of the three scenarios we describe in this study, we estimated the potential impact of block grant funding or per capita caps on the service capacity for health centers in each state. This was done by calculating the necessary reductions in the percentage of nonclinical service full‐time equivalents (FTEs) to maintain the baseline level of clinical service FTEs in a budget‐neutral manner from 2020 to 2024. Our definition of nonclinical service employees included case managers, education specialists, outreach workers, eligibility assistance staff, transportation staff, interpreters, other enabling services personnel, and administrative staff. Clinical staff were defined as medical, dental, mental health, and substance use disorder treatment providers, lab technicians, and other specialists. This demonstrative scenario was informed by our interviews with health center executives, who indicated that they would first start by cutting back on nonbillable staff in the event of revenue shortages due to reduced federal Medicaid contributions. While this is an informative exercise, we recognize that it would be impractical for health centers facing larger cuts to reduce FTEs solely from nonclinical services, given that retention of some nonclinical staff is required to support basic health center operations. Similarly, we did not take into account how the elimination of members of the nonclinical team would affect physician productivity. It would very likely decrease, potentially resulting in lower levels of clinical service even if the clinical FTEs remained at the baseline level.

Given the assumptions built into our models, our findings are conservative and likely underestimate the total reductions in revenues and service capacity that would occur if the changes were to take place.

The scenarios in our study are as follows:

- Baseline scenario (S0): We began by projecting health center patient populations and revenues, separately by payer type, assuming that status quo Medicaid policies and eligibility income limits (as a ratio of FPL) remain unchanged through 2024. Using the baseline estimates from S0 and state‐level population projections, we estimated the percent revenue shortages under the three scenarios described next. Then, to estimate the potential implications of revenue shortages, we projected baseline staffing levels by service type in FTEs, as defined and operationalized by HRSA in the UDS, at the health center level between 2020 and 2024, using projected baseline revenues and service costs under the three scenarios. Analyses were conducted at the state level.
- Phase‐out of EFMAP (S1): All federal bills proposed to eliminate the Medicaid expansion group, in either 2020 or 2024. This simulation model assumed that, starting in 2020, only the federal contribution to Medicaid would decrease; state contributions were held constant to 2016 levels.
- Medicaid block grants (S2): All federal bills would have given states the option to use block grant funding for Medicaid, although the targeted eligibility categories varied across the proposals. This simulation model accounted for the phase‐out of the EFMAP in 2020 and relied on federal contributions computed using 2016 expenditure levels as the baseline and, per the federal proposals, adjusted for inflation, by first using the current CPI‐M and estimated CPI‐Ms, which were derived from a linear model between CPI‐Ms and CPI‐Us, and adopting for future years the CBO's projection of CPI‐Us to predict CPI‐Ms (see the Online Appendix).
- Medicaid Per Capita Caps (S3): All federal bills proposed to convert the Medicaid program financing model to a per capita cap model beginning in 2020, with some inflation adjustment. This simulation model was implemented using 2015‐2016 per capita Medicaid expenditure levels as the baseline and adjusted for inflation in the same way as in S2.

Note that Medicaid expansion enrollees from New Hampshire and Iowa (two of our sampled states) receive subsidies to purchase private qualified health plans (QHPs) through the Health Insurance Marketplace rather than Medicaid coverage; as a result, in these states, health center revenues from the Medicaid expansion population are classified as private plan revenues rather than Medicaid revenues. We therefore excluded New Hampshire and Iowa from the quantitative analyses to avoid skewing revenue projections under different Medicaid rollback scenarios.

#### Statistical Analysis

We used the existing UDS data from 2000 to 2016 to estimate models that predict patient load and health center revenues at the state level, which were subsequently used to simulate outcomes (ie, Medicaid revenue and FTE cuts) for FYs 2020 through 2024 based on future population projections. Under the baseline scenario (S0), we projected patient population levels by fitting linear regression models separately by state and by payer type. For example, the model for estimating the size of the Medicaid patient populations is as follows:

(1) $$\begin{array}{ccc} {P\;\mathrm{atients}}_{s\;,t,\mathrm{Medicaid}} & = & \beta_{1}P_{s\;,t,1-18}E_{s\;,t,\mathrm{Child}}\\ & & +\;\beta_{2}P_{s\;,t,19-49,\mathrm{Female}}E_{s\;,t,P\;\mathrm{arents}}\\ & & +\;\beta_{3}P_{s\;,t,19-64}E_{s\;,t,\mathrm{Adults}}\\ & & +\;\beta_{4}P_{s\;,t,65+}E_{s\;,t,\mathrm{Adults}}+\beta_{5}P_{s\;,t,19-64} \end{array}$$

where *P* is the number of persons in each age population category, *E* is Medicaid eligibility threshold for each population category, *s* indexes states, and *t* indexes years.

Similar models were used to project the Medicare patient population, other publicly insured patient population, privately insured patient population, and uninsured patient population levels (see Online Appendix equations A1 through A4). For the latter two categories, we also included a dummy variable for years 2014 and later to account for the implementation of the Health Insurance Marketplace.

Our revenue projections were similarly computed separately by state and payer source. To address potential lags in Medicaid reimbursements, we smoothed out Medicaid per capita revenues by constructing moving averages with one lead and one lag year. The model used to project per capita Medicaid revenues is as follows:

(2) $$\begin{array}{ccc} \;R_{s\;,t,\mathrm{Medicaid}} & = & \alpha_{0}\;+\alpha_{1}R_{s\;,t-1,\mathrm{Medicaid}}\\ & & +\;\alpha_{2}t+a_{3}t*\mathrm{ACA}+\alpha_{4}t*\mathrm{DRA} \end{array}$$

where *R* represents per capita revenues, *s* indexes states, *t* indexes years, *ACA* is an indicator for expansion states in postexpansion years, and *DRA* is an indicator for years after the Deficit Reduction Act of 2005. Per capita revenues from Medicare, other public insurance, private insurance, and self‐pay (ie, payments from uninsured patients) were projected using similar models (see Online Appendix equations A5 through A8).

Finally, to estimate baseline FTE levels, we first projected the cost of service FTEs (accounting for both staff compensation and equipment depreciation) at the health center level, separately by state and service type. For example, the model to estimate medical FTE costs is as follows:

(3) $$\begin{array}{ccc} C_{s\;,t,\mathrm{Medical}} & = & \beta_{0}\;+\left(\beta_{1,s}\mathrm{Physician}\_{\mathrm{FTE}}_{\mathrm{HC},t}\right.\\ & & \left.+\;\beta_{2,s}\mathrm{APC}\_{\mathrm{FTE}}_{\mathrm{HC},t}+\beta_{3,s}\mathrm{Other}\;\mathrm{Med}\_{\mathrm{FTE}}_{\mathrm{HC},t}\right)\\ & & *\;{\left(1+r_{s}\right)}^{t} \end{array}$$

where *C* represents service‐category FTE costs, *s* indexes states, *HC* indexes health centers, *t* indexes years, *r* represents labor cost growth, *APC* represents advanced practice clinicians (nurse practitioners, physician assistants, certified nurse midwives), and *OtherMed* includes nurses and other medical, laboratory, and x‐ray personnel. We estimated similar models for FTE costs of mental health, dental, administrative/enabling and other services (see Online Appendix equations A9 through A12). Then, by assuming that health centers continue to allocate revenues to each service category by the same proportions as in 2016, we estimated baseline FTEs by service category for 2020 through 2024 by dividing the projected revenues allocated to each category by the projected FTE costs.

To project revenue shortages under different Medicaid reform scenarios, we began by disaggregating total projected revenues under the baseline scenario (S0) into the state and federal shares of contributions based on FMAP rates specified in the ACA. Under S1 (phase‐out of EFMAP), federal contributions were reduced by the difference between EFMAP and traditional FMAP in 2016 multiplied by the projected number of Medicaid expansion enrollees for each state during 2020‐2024. Under S2 (block grant), the total federal reductions in 2020‐2024 equaled the difference between the projected baseline (S0) federal contributions and the 2016 contributions for the traditional population adjusted by an annual inflation factor of 2.3% (ie, projected contributions under S2). Finally, under S3 (per capita cap), federal per capita contributions to each state in 2020 were estimated by adjusting 2015‐2016 per capita contribution levels for the traditional population first by an annual inflation factor of 2.3%, and then by the percent deviance of each state's projected per capita expenditures from the national average projected PC expenditures from the previous year. Federal per capita contributions for 2020‐2024 were subsequently forecasted dynamically based on projected contributions from the prior year. Finally, total annual federal Medicaid contributions reductions under S3 were computed by subtracting the product of the projected S3 per capita contributions and projected number of Medicaid enrollees from the total projected baseline (S0) level contributions for each state.

For each scenario, we estimated the percentage of nonclinical service FTE reductions necessary to maintain the baseline level of clinical service FTE in a budget‐neutral manner from 2020 to 2024. These estimates were computed by assuming that (a) there would be no cuts to Medicaid enrollment (ie, all revenue shortages would be absorbed by health centers through cuts to services and/or provider reimbursement rates), or (b) Medicaid enrollment would be reduced in proportion to revenue shortages (such that some Medicaid revenue losses would be offset by private insurance or self‐pay revenues as patients were transitioned from Medicaid to the health centers’ sliding‐fee scale).

Sensitivity analyses were also conducted for each projection, assuming various levels of Medicaid disenrollment (relative to baseline) in expansion states, which were derived from information collected from state waiver documents. Disenrollment ranged from a low of 5% to a maximum of 30% and was examined in 5 percentage‐point increments.

### Qualitative Analysis

*Document Review of Key Elements of §1115 Waivers in Seven Targeted States*. As background for our second aim/research objective, we conducted a document review of all applicable Medicaid documents for seven states with pending or approved §1115 waivers that imposed one or more of the changes that aim to restrict enrollment into the program: Arizona, Arkansas, Iowa, Indiana, Kentucky, Montana, and New Hampshire. We identified official waiver application and CMS correspondence documents from the CMS website and official state Medicaid websites. For each state, we reviewed the original or modified waiver application; approval, terms, and conditions (if approved); fact sheets; and any amendments. As of May 2018 (when we concluded the qualitative analysis of our study), waivers in Arkansas, Iowa, Indiana, Kentucky, and Montana had been approved, and waivers in Arizona and New Hampshire were still pending. Arkansas had started implementing the changes, whereas Kentucky's waiver implementation had been stopped by court proceedings. All of these states are Medicaid expansion states (Iowa and New Hampshire have waivers to use Medicaid as a subsidy for insurance products purchased on the state marketplace).

Although many of the details of the waivers differ across states, the contents of each waiver can be generally categorized by the type of requirements (eg, imposition of premiums or cost sharing and conditions for eligibility such as work or healthy behavior requirements) and eligibility lockout periods. Understanding these different facets of each state's waiver proposal guided our methodological approach, both in informing the assumptions of the simulation model, and especially in guiding the collection and analysis of our qualitative interview data.

*Semistructured Interviews of a Purposeful Sample of Health Center Leaders in Seven Targeted States*. We reached out to chief executive officers and chief financial officers of health centers located in the seven selected states to conduct semistructured interviews, with the goal of speaking to three to five health center executives in each state. We identified health center and chief executive officer contact information from the 2016 UDS data and began outreach with a small sample of health centers from each state, selected using a purposeful sampling approach to maximize diversity in health center size (both in terms of number of patients and number of sites), geography, and rural vs urban locality. Our initial sampling approach excluded health centers that did not provide any of the following four services: dental, vision, mental/behavioral, and substance use disorder treatment. We invited health center leaders by email to participate in a brief, confidential interview and followed up (also by email) with those who did not respond up to two times, with at least one week lag time in between each contact. When response rates were inadequate, we expanded our outreach sample gradually until we had reached out to close to the full universe of health centers in a number of states. We reached our interview target sample in all but one state (Arkansas, *n* = 2).

Based on the information reported in the 2016 UDS data, our sample of health centers (*n* = 23) was diverse. Approximately 40% of our sample was located in urban counties and 40% was in rural counties; 60% had fewer than 15 sites in 2016. All but one health center had at least one mental health FTE, while only one health center had at least one FTE for vision services. There was also a good distribution in health center size by patient population in our interviewed sample, with 33% of the health centers providing services to up to 10,000 patients, 40% providing services to up to 30,000 patients, and the remainder providing services to over 30,000 patients. At approximately half of the health centers sampled, 50% of patients were from households with incomes below 150% of FPL. Finally, based on interview data, we found that Medicaid was the primary payer for 26% to 75% of patients served at 90% of health centers for which we obtained payer‐mix information (*n* = 18).

Interviews were conducted between March and May 2018, and most were conducted over the phone. However, because our study timeline coincided with the National Association of Community Health Centers Policy and Issues Forum in Washington, DC, we also took the opportunity to conduct in‐person interviews with some attendees.

Interviews lasted between 20 and 45 minutes and focused on the following:

- The interviewee's understanding of how their state‐specific waivers may affect the patient population and operations at their health center;
- The participant's perception of how federally imposed block grants or per capita caps may affect their health center specifically;
- Whether the participant's health center had begun taking any concrete actions to prepare for the potential impacts of these changes; and
- The direction of the Medicaid program that the interviewee anticipated their state may take given increased flexibility.

Interviews were not tape‐recorded to protect participant confidentiality. With few exceptions, each interview was conducted by at least two researchers, with one person leading the interview, and the other(s) taking close notes (including verbatim notes) of the participants’ responses (with any identifying information redacted).

## Results

### Descriptive Statistics

Table 1 presents overall summary statistics of the analytical dataset at three points in time. The total number of health centers in the 50 states and District of Columbia grew from 703 in 2000 to 1,337 in 2016. Within our analytical sample, the total health center patient population grew from 9.1 million to 25.4 million over the same period, with the total Medicaid‐covered health center patient population growing from 2.9 million (32%) in 2000 to 12.4 million (49%) in 2016. Accordingly, health centers nationwide received approximately $1.3 billion in Medicaid revenues in 2000 and $10.1 billion in 2016 for services provided to their Medicaid‐covered patient populations. Total health center service capacity in staff FTEs grew more than four‐fold between 2000 and 2016; the proportion of mental and dental service FTEs increased, while the proportion of administrative FTEs decreased.

**Table 1:** National Overview of the Study Analytical Sample, 2000, 2012, and 2016

	2000		2012		2016	
	Total	% of total^a^	Total	% of total^a^	Total	% of total^a^
No. of health centers	703	–	1,169	–	1,337	–
***No. of health center patients (in thousands)***						
Total	9,090	–	20,658	–	25,408	–
Medicaid	2,923	32%	8,133	39%	12,448	49%
Medicare	650	7%	1,671	8%	2,354	9%
Private insurance	1,429	16%	2,903	14%	4,388	17%
Self‐pay	3,762	41%	7,466	36%	5,963	23%
Other public insurance	313	3%	517	3%	255	1%
***Annual health center revenues (in thousands***)						
Total	$3,695	–	$14,227	–	$22,471	–
Total patient revenues	$2,081	–	$8,893	–	$15,250	–
Medicaid patients	$1,290	62%	$5,626	63%	$10,108	66%
Medicare patients	$204	10%	$874	10%	$1,685	11%
Other publicly insured patients	$268	13%	$1,085	12%	$2,233	15%
Private insurance patients	$223	11%	$918	10%	$1,007	7%
Self‐pay patients	$96	5%	$377	4%	$249	2%
Grants	$1,614	–	$5,334	–	$7,221	–
***Health center provider capacity (in FTEs)***						
Total	53,707	–	152,323	–	219,178	–
Medical	19,417	36%	53,555	35%	73,602	34%
Mental health	1,131	2%	5,808	4%	10,176	5%
Dental	2,688	5%	11,535	8%	16,554	8%
Other professional services^b^	16,930	32%	59,052	39%	83,871	38%
Substance abuse	498	1%	920	1%	1,310	1%
Nonclinical^c^	13,024	24%	21,429	14%	33,687	15%

Abbreviations: FTE, full‐time equivalent; HC, health center.

a For HC revenues, % of total represents the percentage of total patient revenues.

b Other professional services personnel includes audiologists, podiatrists, physical therapists, etc.

c Nonclinical personnel includes administrative and enabling staff.

Table 2 compares average state‐level health center patient load, revenues, and service FTEs for expansion vs nonexpansion states. Notably, even though nonexpansion states have a larger population below 150% FPL, the percentage of health center patients with Medicaid coverage has historically been higher in Medicaid expansion states, and the difference increased substantially between 2000 (34% vs 27%) and 2016 (55% vs 33%). Much of the difference in Medicaid patient population size between expansion and nonexpansion states is accounted for by self‐pay (ie, uninsured) patients. From 2000 to 2016, the percentage of self‐pay patients at health centers was much higher in nonexpansion states than in expansion states (47% vs 39% in 2000; 36% vs 19% in 2016).

**Table 2:** Comparison of Expansion vs Nonexpansion States in the Study Analytical Sample, 2000, 2012, and 2016

	2000				2012				2016			
	Expansion		Nonexpansion		Expansion		Nonexpansion		Expansion		Nonexpansion	
	(*n* = 32)^a^		(*n* = 19)^a^		(*n* = 32)^a^		(*n* = 19)^a^		(*n* = 32)^a^		(*n* = 19)^a^	
	Mean (SD)	% of Total	Mean (SD)	% of Total	Mean (SD)	% of Total	Mean (SD)	% of Total	Mean (SD)	% of Total	Mean (SD)	% of Total
***State population (in millions)***												
Total	5.6 (6.74)	–	5.4 (5.2)	–	6.1 (7.3)	–	6.3 (6.5)	–	6.2 (7.5)	–	6.5 (6.9)	–
<150% FPL	0.84 (1.2)^b^	15%^b^	0.9 (1.0)^b^	17%	1.4 (1.9)	24%	1.6 (1.8)	26%	1.2 (1.7)	20%	1.5 (1.6)	22%
**Average no. of health centers per state**	14	–	14	–	24	–	22	–	28	–	24	–
***No. of health center patients (in thousands)***												
Total	195 (248)	–	150 (124)	–	452 (612)	–	326 (304)	–	566 (818)	–	384 (371)	–
Medicaid	67 (91)	34%	41 (35)	27%	193 (272)	43%	103 (108)	32%	313 (520)	55%	128 (139)	33%
Medicare	12 (14)	6%	14 (10)	9%	35 (37)	8%	29 (22)	9%	51 (57)	9%	38 (28)	10%
Other public insurance	8 (19)	4%	3 (4)	2%	12 (29)	3%	7 (17)	2%	5 (11)	1%	5 (14)	1%
Private Insurance	31 (33)	16%	23 (15)	15%	64 (60)	14%	45 (28)	14%	92 (88)	16%	76 (60)	20%
Self‐pay	76 (104)	39%	70 (71)	47%	149 (239)	33%	142 (148)	44%	105 (166)	19%	137 (146)	36%
***Annual health center revenues (in thousands)*** c												
Total patient revenues	$49 (70)	–	$27 (22)	–	$212 (304)	–	$111 (105)	–	$378 (639)	–	$166 (163)	–
Medicaid patients	$32 (49)	65%	$14 (13)	52%	$139 (215)	66%	$62 (69)	56%	$266 (510)	70%	$84 (101)	51%
Medicare patients	$4 (6)	8%	$4 (3)	15%	$19 (24)	9%	$14 (11)	13%	$39 (55)	10%	$23 (18)	14%
Other publicly insured patients	$3 (6)	6%	$0 (1)	0%	$10 (31)	5%	$3 (8)	3%	$6 (15)	2%	$3 (9)	2%
Private insurance patients	$6 (6)	12%	$4 (3)	15%	$25 (24)	12%	$15 (10)	14%	$49 (50)	13%	$35 (30)	21%
Self‐pay patients	$4 (6)	8%	$5 (5)	19%	$18 (23)	8%	$18 (17)	16%	$19 (21)	5%	$21 (19)	13%
Grants	$35 (43)	–	$26 (27)	–	$118 (155)	–	$82 (80)	–	$155 (207)	–	$119 (124)	–
***Health center provider capacity (in FTEs)***												
Total	1217	–	777	–	3443	–	2218	–	5045	–	3039	
Medical	415 (540)	34%	323 (280)	42%	1179 (1570)	34%	833 (776)	38%	1660 (2442)	33%	1078 (1042)	35%
Mental health	30 (60)	2%	9 (12)	1%	134 (159)	4%	80 (105)	4%	242 (327)	5%	128 (168)	4%
Dental	65 (540)	5%	32 (280)	4%	253 (1570)	7%	181 (776)	8%	376 (2442)	7%	238 (1042)	8%
Substance abuse	12 (27)	1%	6 (12)	1%	24 (43)	1%	8 (12)	0%	35 (52)	1%	10 (11)	0%
Nonclinical^d^	402 (834)	33%	214 (184)	28%	1349 (1909)	39%	836 (729)	38%	1934 (2705)	38%	1157 (1213)	38%
Other professional services^e^	293 (464)	24%	192 (249)	25%	504 (750)	15%	279 (260)	13%	798 (1305)	16%	429 (429)	14%

Abbreviations: FPL, federal poverty level; FTE, full‐time equivalent; HC, health center.

a Expansion status as of March 2018. Expansion group includes Washington, DC.

b Population <150% FPL data are not available for 2000. The 2000 figures reflect population <125% FPL.

c For HC revenues, % of total represents the percentage of total patient revenues.

d Nonclinical personnel includes administrative and enabling staff.

e Other professional services personnel includes audiologists, podiatrists, physical therapists, etc.

The trend in Medicaid revenues as a share of total health center patient revenues mirrors the trend in the share of health center Medicaid patients. In 2000, Medicaid revenues accounted for 65% of health center revenues in states that would expand Medicaid and 52% in states that would not; in 2016, the percentages were 70% in expansion states and 51% in nonexpansion states.

Finally, the number of medical providers serving health centers at the state level also grew faster in expansion states than in nonexpansion states. In expansion states, the number increased by more than 300%, from an average of 415 FTEs in 2000 to an average of 1,660 FTEs in 2016. By comparison, there was nearly a 234% increase in nonexpansion states, from an average of 323 FTEs in 2000 to an average of 1,078 FTEs in 2016. Similar patterns were observed for substance use disorder treatment provider FTEs. Conversely, mental health, dental, and administrative FTEs grew faster on average in nonexpansion states; most notably, mental health provider FTEs grew over 1,300% in nonexpansion states and just over 700% in expansion states between 2000 and 2016, although absolute levels still remained much lower in nonexpansion states.

### Simulation Results

**Figure 2:**
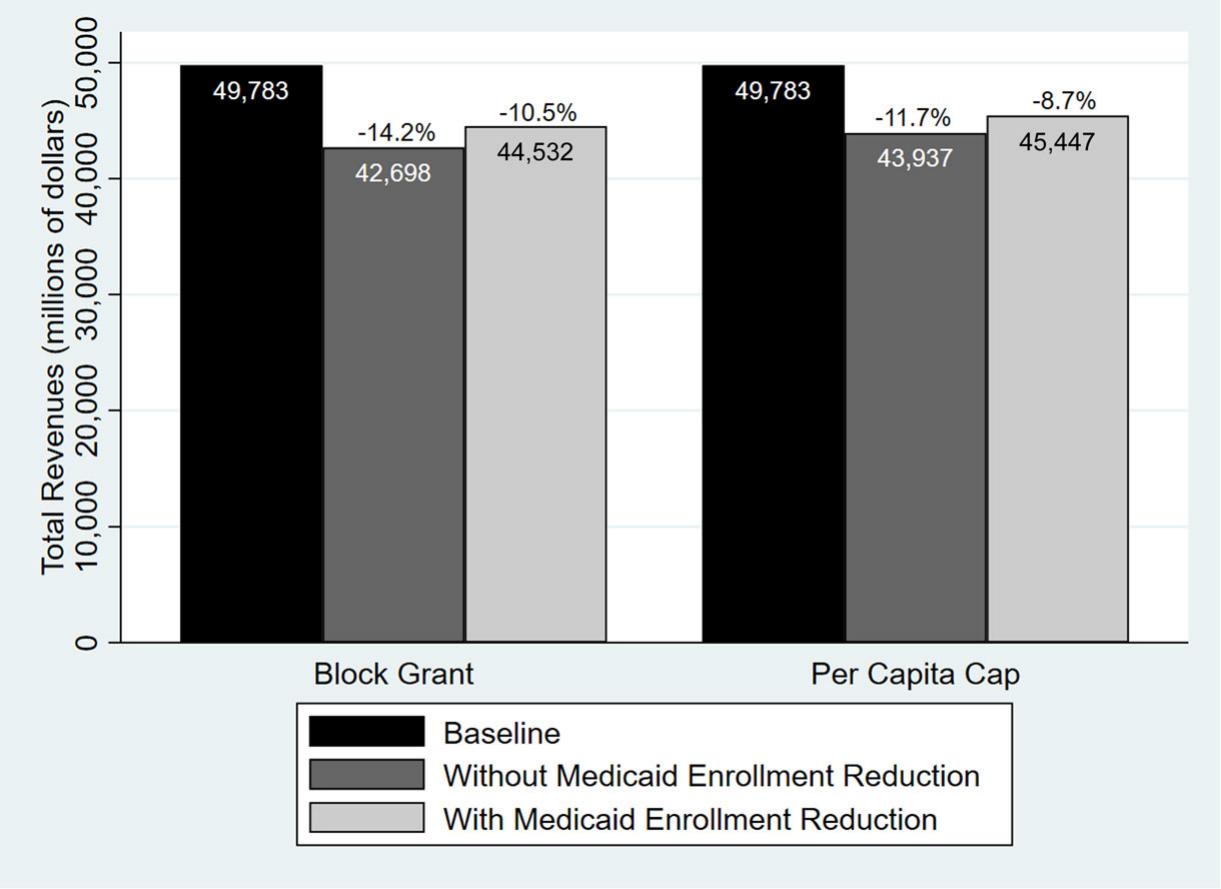
Comparison of Projected Total Annual Gross Health Center Revenues Under the Baseline, Block Grant, and Per Capita Cap Scenarios in 2024 [Color figure can be viewed at http://wileyonlinelibrary.com]

**Figure 3:**
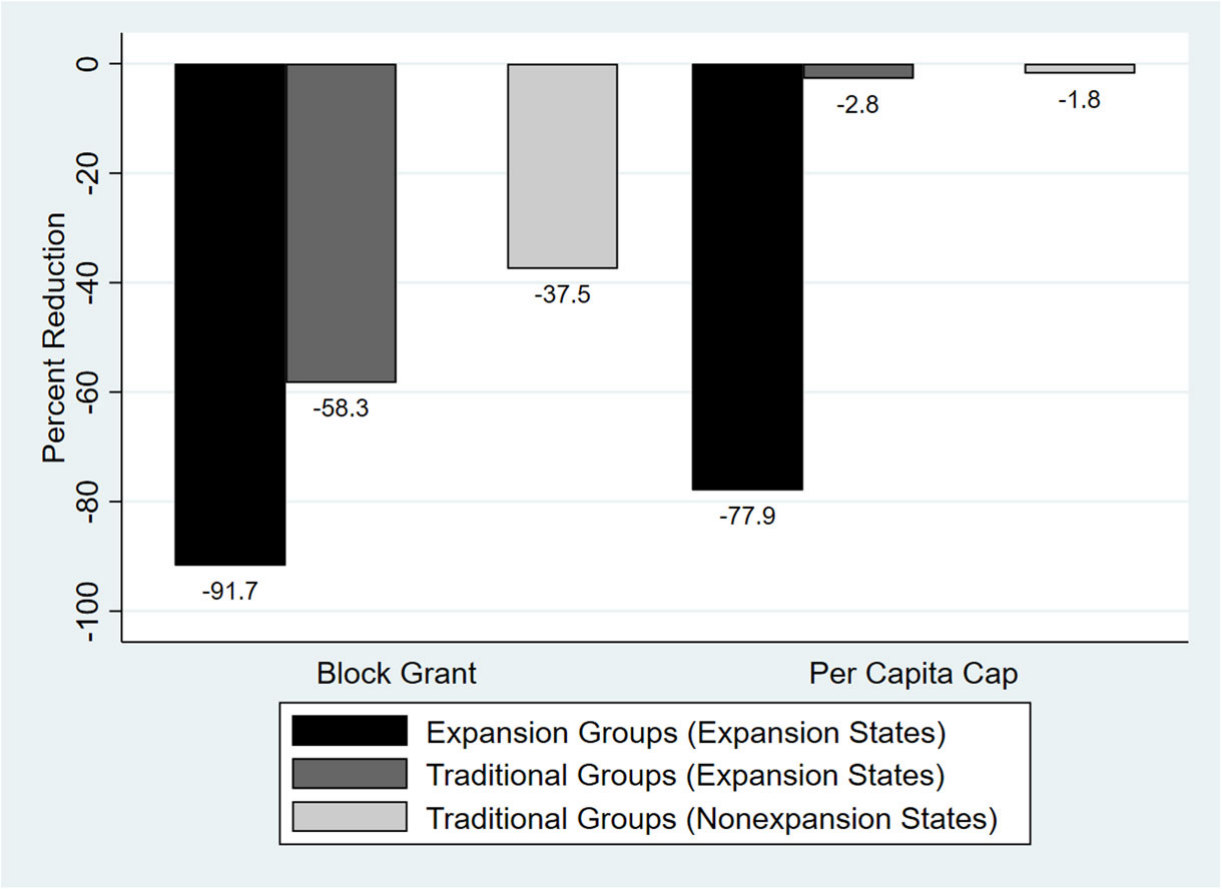
Gross Revenue Changes in 2024 as a Percentage of Baseline Projected Revenues Under the Block Grant and Per Capita Cap Scenarios: Traditional vs Expansion Coverage Groups [Color figure can be viewed at http://wileyonlinelibrary.com]

**Figure 4:**
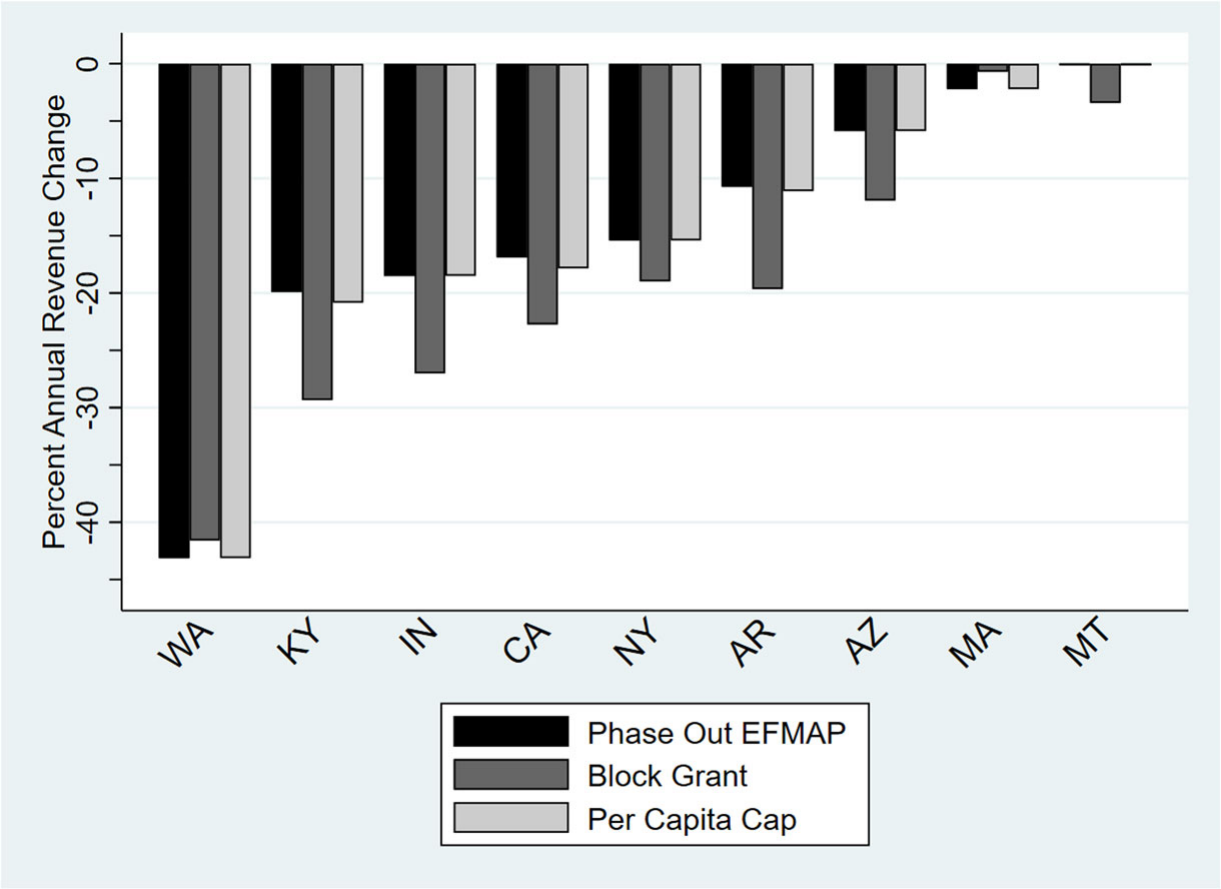
Projected Impact of Proposed Federal Medicaid Changes on Total Health Center Revenues in Selected Expansion States by 2024^a^ ^a^N = 405 health centers across the eight states in 2016. Expansion status is as of March 2018. Washington is included in the graph because it is the *most* affected state. California, New York, and Massachusetts are included because they are highly populated states, and California and Massachusetts have relatively generous Medicaid programs. The other states (Kentucky, Indiana, Arkansas, Arizona, and Montana) are part of the study sample. New Hampshire and Iowa are part of the sample but were excluded from the graph because they expanded via private plan subsidies (so expansion revenues are recorded as private revenues). [Color figure can be viewed at http://wileyonlinelibrary.com]

Both per capita caps and block grants would result in a reduction in total health center revenues. At a minimum, cuts in revenues in 2024 would average approximately 11% under a block grant scenario and 9% under a per capita cap scenario across all states (Figure 2). In terms of actual dollars, these percentage cuts represent decreases between $5.3 billion and $7.1 billion for all states under a block grant scenario, and a $4.3 billion to $5.8 billion decrease under a per capita cap scenario. In either scenario, the revenue impact would be greater for health centers in expansion states, even among traditional enrollee groups, than in nonexpansion states (Figure 3). Figure 4 displays the estimated reductions in annual total revenues by 2024 in the most‐ and least‐affected expansion states, including five of the seven states we targeted for focused analyses and interviews (Arizona, Arkansas, Indiana, Kentucky, and Montana).

Revenue changes vary widely in each scenario and by state. Health centers in the most‐affected expansion state, Washington, would see a revenue cut of over 40% compared to baseline projections; in Montana, the least‐affected state among Medicaid expansion states, a cut of 0% to 3% was predicted. Other states that consistently fall within the top third of the most‐affected states across the three policy scenarios are Kentucky, Indiana, Rhode Island, Ohio, California, New York, Pennsylvania, and New Mexico; the least‐affected states, with revenue cuts ≤5% of baseline revenues across the three scenarios, are Montana, Alaska, Maryland, Illinois, Louisiana, Massachusetts, West Virginia, and New Jersey (see Online Appendix Table 1a).

**Figure 5:**
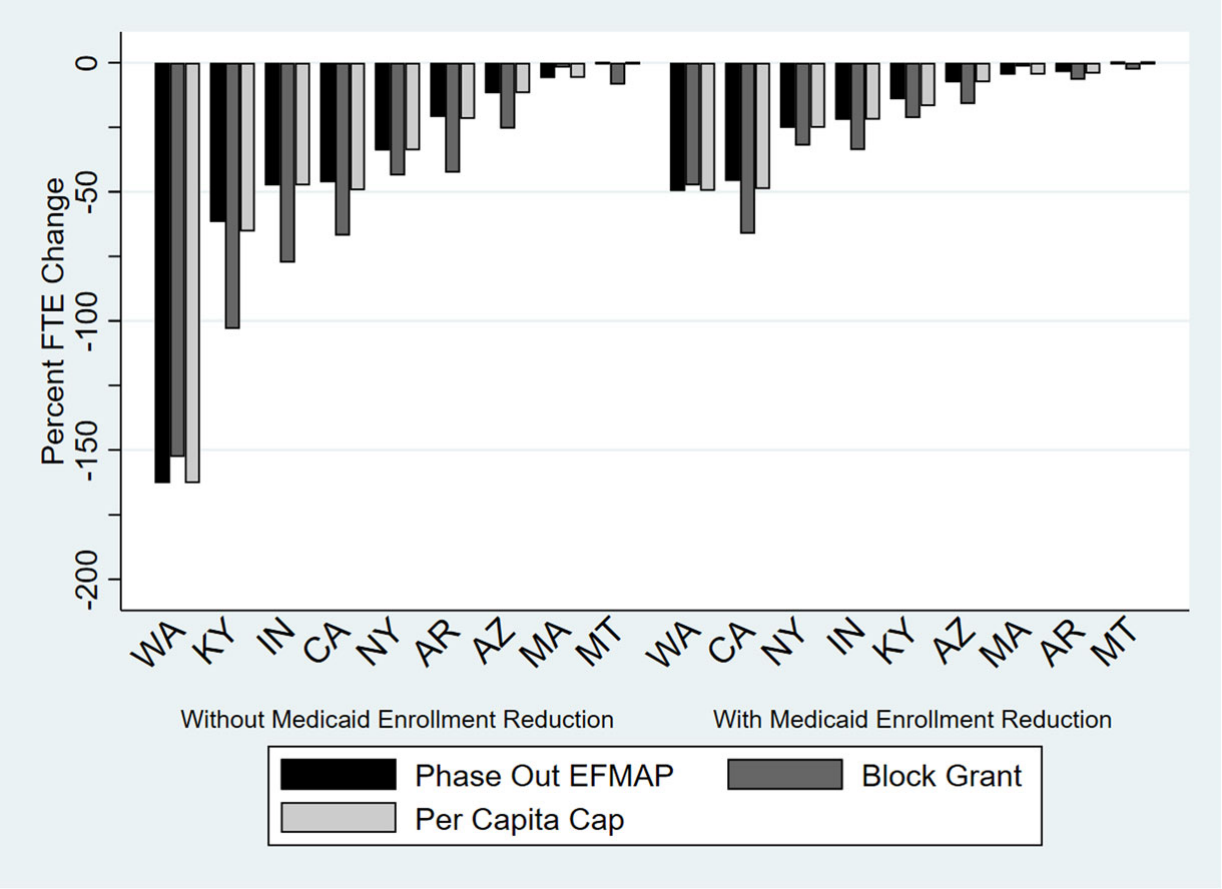
Changes in Nonclinical FTEs to Maintain Baseline Level of Clinical FTEs in Selected Expansion States by 2024^a^ ^a^N = 405 health centers across the 8 states in 2016. Expansion status is as of March 2018. [Color figure can be viewed at http://wileyonlinelibrary.com]

For illustrative purposes, we modeled how the simulated revenue changes would affect nonclinical FTEs if health centers were to maintain baseline clinical FTE levels while remaining budget neutral (Figure 5 and Online Appendix Tables 2a and 2b). This scenario was informed by our qualitative findings that, in the event of revenue shortages, health centers would likely reduce nonclinical (eg, administrative and enabling) FTEs before cutting clinical FTEs. While our simulated scenario is extreme, and would be unrealistic for health centers facing larger cuts, the results are informative for illustrating the variation in impacts. Notably, assuming no Medicaid enrollment reductions, total revenue reductions would be so great in Washington state that health centers would need to roll back administrative/enabling FTEs by more than 150% to maintain clinical FTEs under all three federal change scenarios. In other words, health centers would, on average, not be able to maintain their baseline level of clinical service FTEs in a budget‐neutral manner even if all nonclinical FTEs were eliminated. Additionally, in line with trends in revenue shortages across expansion states, Washington, Kentucky, Indiana, Rhode Island, California, New Mexico, Ohio, New York, and Pennsylvania would need to reduce nonclinical FTEs most significantly, whereas Montana, Illinois, Arkansas, Maryland, Louisiana, Massachusetts, West Virginia, and New Jersey would need to reduce nonclinical FTEs marginally to maintain clinical FTEs and budget neutrality across scenarios (see Online Appendix Tables 2a and 2b).

The simulation results assuming no Medicaid enrollment reductions represent the upper bounds of the likely impacts of each federal proposal, and results from simulations assuming that Medicaid enrollment is reduced proportionately to federal contribution reductions represent the lower bounds of the likely impacts. In setting the lower bounds, we anticipated that health centers would recover some Medicaid revenue losses as beneficiaries transitioned to other insurance plans or became self‐paying patients. In the latter case, health centers in Washington state would have to cut their nonbillable FTEs by approximately half to maintain the same level of clinical FTEs; in other states (eg, Montana, Maryland, Louisiana, Massachusetts, and West Virginia), health centers would need to make almost no cuts to nonclinical (and also often nonbillable) FTEs.

**Figure 6:**
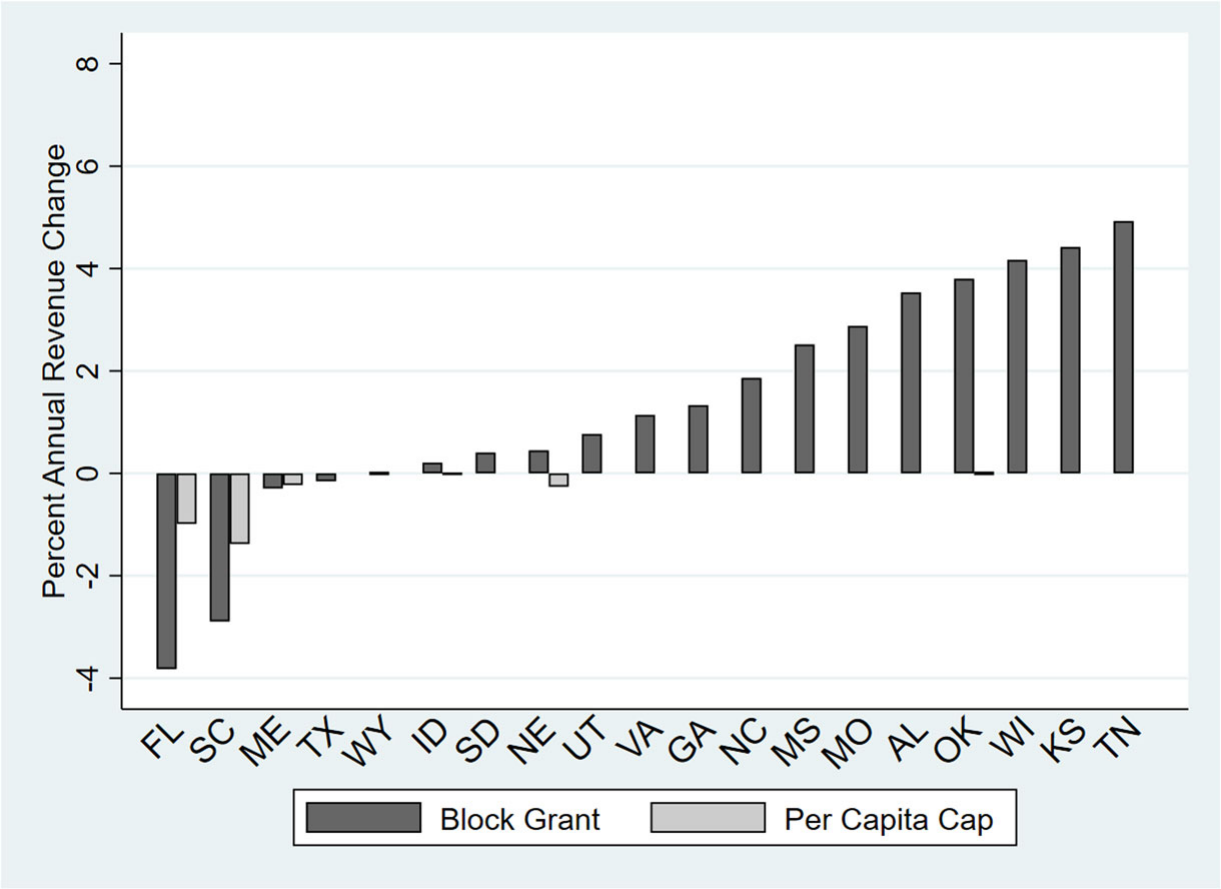
Projected Impact of Proposed Federal Medicaid Changes on Total Health Center Revenues in All Nonexpansion States by 2024^a^ ^a^N = 452 health centers across the 19 states in 2016. Expansion status is as of March 2018. [Color figure can be viewed at http://wileyonlinelibrary.com]

**Figure 7:**
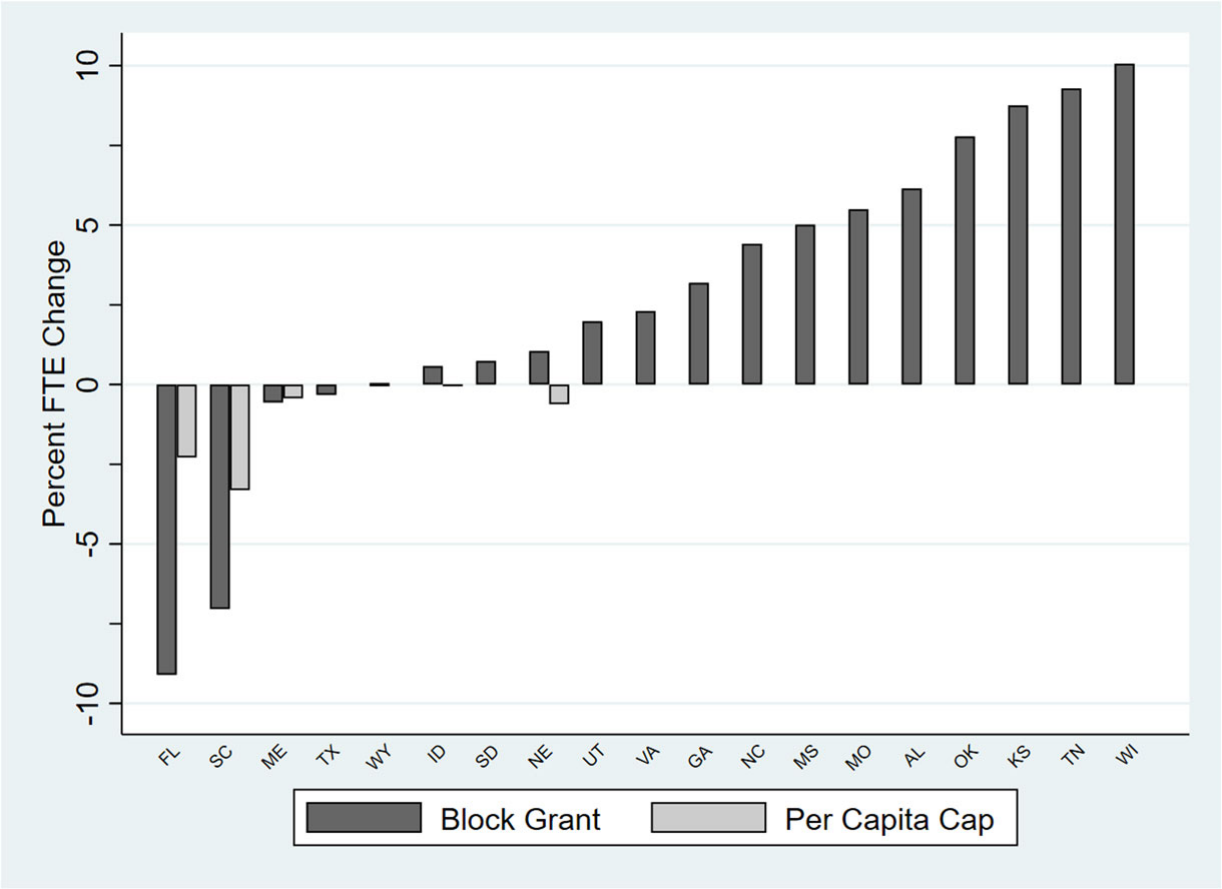
Change in Nonclinical FTEs to Maintain Baseline Level of Clinical FTEs in All Nonexpansion States by 2024^a^ ^a^N = 452 health centers across the 19 states in 2016. We assumed no cuts to enrollment among traditional Medicaid enrollees in nonexpansion states. Expansion status is as of March 2018. [Color figure can be viewed at http://wileyonlinelibrary.com]

Contrast these results with those of health centers in nonexpansion states. Because nonexpansion states do not have an EFMAP, health center revenues would not be affected under S1. Health center revenues in most nonexpansion states also would not be affected under S2 or S3, with only Florida and South Carolina facing notable revenue declines in either scenario (see Figure 6 and Online Appendix Table 1b). Notably, even the largest cut of 4% in the most affected state, Florida, pales in comparison to the projected >40% cut in Washington. A block grant would result in a surplus in the short term that would allow for more nonclinical service FTEs in 14 of the 19 nonexpansion states without compromising clinical FTEs or budget neutrality relative to the baseline scenario by 2024 (see Figure 7 and Online Appendix Table 2b). This is reflective of both the lack of impact of the block grant on total revenues in these particular states and the projected decline in the number of Medicaid patients (eg, Mississippi's Medicaid population was 90,704 in 2016 and is projected to be 85,536 by 2024). This phenomenon is not observed under the per capita cap scenario because federal Medicaid contributions would be determined by the total number of state Medicaid enrollees each year. For the 16 states that are projected to receive baseline levels of Medicaid revenue reductions under a per capita cap, no changes in nonclinical FTEs would be required of health centers to maintain clinical FTEs.

Across the states that we sampled for health center leader interviews (excluding Iowa and New Hampshire because expansion populations in those states received subsidies for private QHPs), projected reductions in total revenue from a federal block grant compared to baseline under the various scenarios would range from 0%‐3% in Montana to 21%‐29% in Kentucky (the second highest revenue loss after Washington state among all states). When it is assumed that health centers would recoup some of their Medicaid losses with revenues from sliding‐fee‐scale patients, projected FTE cuts range from 0%‐3% in Montana to 14%‐21% in Kentucky. In contrast, when it is assumed that health centers would see the same volume of Medicaid patients but need to cut either the scope of services or payments to providers, the projected FTE cuts range from 0%‐8% in Montana to 62%‐103% in Kentucky.

### Qualitative Results

Various health center leaders we interviewed described block grant funding as “a disaster,” “a big step back,” or “devastating,” drawing on their own experiences with other social programs funded through block grants. One executive described block grants as “another form of cost‐shifting [as] the cost of taking care of these folks is shifted to hospitals in uncompensated care and health centers that have to take care of everyone and concluded that “overall population health will suffer.” Another participant anticipated that, “even a shift of 1% of patients from Medicaid to [the] sliding‐fee program has huge consequences [for the health center] … $1 million consequence easily.”

For other executives, the consequences seemed less clear. “It really depends in the end how many people lose their coverage and how many go back to the uninsured category with a sliding‐fee schedule,” one interviewee stated.

Many participants noted that they expected block grants would ultimately harm the quality of care because of their effect on the health centers’ capacity to provide comprehensive care or maintain the “scope or number of locations or number of staff.” As one executive explained, “What we have learned over the past years is that you're always playing catch‐up [with block grants].” Other health center leaders expressed similar sentiments, with one stating, “We have seen what's happened with block grants in other areas—it has not been a positive impact, especially in regions where the population is growing and there's competition between healthcare entities.”

The extent of the impact would vary depending on the size of the health center. According to one interviewee,

The biggest ones will survive—they can absorb some of the lumps and bumps. But a smaller one with a high proportion of uninsured, they're going to have a much harder time. There are community health centers that operate in the death zone … if they have an unanticipated $5k expense, they have a hard time covering that.

While our simulation results show that a per capita cap scenario would result in a range of short‐term losses similar to those from a block grant scenario in most states, health center leaders were generally uncertain about what to expect with a per capita cap approach, since it is a new model with many unknowns. “The cap is essentially unknown. It's scary and gives us anxiety because it would be a completely new model,” one interviewee said. The participants had many questions, such as “What is the baseline?” and “How equitable would the cap be, factoring in the cost of living in different states?”

Several participants emphasized that the Medicaid program under a block grant scenario would no longer be able to respond to changes in demand or other unexpected events that may drive up costs, such as natural disasters, economic downturns, the introduction of a breakthrough drug, or even a severe flu season. Regarding natural disasters, one participant said,

If block grants go through, there's no backup. … The money that we would've had access to under the current system we wouldn't have. You end up with a lot of people with no coverage. We're going to see a lot of sick people lose services. … At the end of the day, the state won't be able to make up those dollars.

Another interviewee focused on a potential recession:

As I understand it, there's no real‐time adjustment for those block grants; it's just going to be set at the federal level—if they give you, say, $1 million and you have 10,000 people on Medicaid, [but] then suddenly you get a recession and that number goes up—now you have 20,000 with the same $1 million. … There needs to be some safety mechanisms built into that.

One participant suggested that the impact of a block grant could be lessened if it were designed to include a sustainable rate increase or tied to some sort of inflation target that corresponds with health care inflation.

Interviewees also raised concerns about how a block grant approach would shift greater decision‐making responsibility to the state level. One leader expressed skepticism about the extent to which state governments would be adequately able to handle the new responsibility:

As crazy as the show gets in DC, it's a more stable one than here. State politics is a lot more ebb and flow—not a lot of consistency. If you get some sort of state financing for something, you can never depend on it for the next year and plan; it's so volatile.

Another participant observed, “States will follow history and do the least common denominator. State revenue won't be there to make up services and then they will be asking for federal dollars. Asking states to do this will create a lot of turmoil and will definitely increase the uninsured rate.” Some interviewees said that their states would at least try to find alternative sources of funding. For example, one participant said, “The general assembly will make some attempt to make up some of it, but they won't be able to make up all of it.”

Interestingly, while advocates of block grants view the increased degree of state decision‐making regarding the Medicaid program as a desirable driver of innovation, the health center leaders we interviewed seemed less optimistic. One participant noted that “the notion of block granting is all about cost containment” and that state flexibility and innovation are “bogus arguments.” This interviewee further explained, “We already have all the flexibility, we just haven't had enough resources—it's really undermined innovation.”

Some were similarly concerned about the types of decisions their states would make, such as restricting Medicaid eligibility and funneling the funds to other purposes. For example, one health center leader stated, “I know how this state would function—they wouldn't want to open it up to all children, they would skim off the top.” Another said, “It would be detrimental to our whole state. Once it [Medicaid funding] becomes a block grant, Congress could allocate these funds to something else. Anything in a rural state can be considered health‐related: roads, bridges. Money would be automatically allocated to things like that—infrastructure.”

A few participants also expressed that if block grants were implemented, their state would try to tweak the existing Medicaid program, and the directions currently taken under new §1115 waivers are good indications of the directions that states might take with the block grant program. According to one interviewee, “The proposed waiver was the preemptive strike—set up some sort of regional infrastructure so that if a block grant goes through, everything will be set up.” Similarly, another participant said,

If we end up with block granting Medicaid, and especially with other state budget pressures we're feeling around here, they'll just go further with doing away with Medicaid. That's their goal. Their goal is to get people off it—they see it as an entitlement program—[and] get people into good paying jobs with good benefits that don't exist.

Finally, the overwhelming sentiment shared among our interviewees was the concern around the detrimental impacts that federal‐ and state‐level Medicaid cutbacks would ultimately have on population health over the long term. Compounding the consequences of reduced health center service capacities, a number of health center leaders said they anticipate that many patients who lost Medicaid coverage would become less likely to seek necessary care in a timely manner, thus worsening health outcomes and escalating costs further down the line. Emphasizing that “prevention and care along the way is so much more cost‐effective in the long run,” one participant described efforts to reduce Medicaid spending as “short‐sighted.”

## Discussion and Implications

Our simulation results show that block grants or per capita caps would have a detrimental effect on total health center revenues, with cuts relative to baseline projected to reach at least 11% (approximately $5.3 billion) for block grants and 9% (approximately $4.3 billion) for per capita caps overall, across all states, by 2024. Across expansion states, the resulting cuts in nonclinical FTEs needed to maintain baseline medical care would range from 0% to 163% under a per capita cap scenario that assumes proportional rollbacks in Medicaid enrollment; nonclinical FTE cuts in these states would be between 1% and 153% under a block grant scenario that assumes no reductions in Medicaid enrollment. The impacts in nonexpansion states are projected to be substantially smaller. Some nonexpansion states would not be negatively affected under block grants or would break even under per capita caps *in the short term*. The direction of these simulated impacts are in part due to population projections over the study period and the existing structure of the Medicaid program in those states; as noted previously, they do not account for a possible recession or other unanticipated or catastrophic events.

As explained in the methods section, our simulation models did not take into account a number of factors that could impact total health center revenues. These factors include other potential legislative changes that reduce state funding levels or federal grant funding, or otherwise contribute to potential future funding cliffs for health centers; their negative effects would be expected to compound those of Medicaid block grants or per capita caps. Additionally, our models did not address potential changes in health center behaviors or the complex dynamics involving insurance status, patient behaviors, the demand for and use of health center services, and potential changes to population health. Thus, our results should be interpreted in the context of these factors as well as the current legislative environment.

In our interviews, health center leaders discussed specific impacts that they anticipate decreased Medicaid revenues would have on primary care for Medicaid patients and underscored the challenges of operating in a block grant environment. Overall, the interviews demonstrated that health centers are facing a “tremendous amount of uncertainty” with regard to the Medicaid landscape, which hampers their ability to plan and prepare for future changes. For example, some health centers have temporarily halted plans to build new sites or hire new staff. Although congressional action to repeal and replace the ACA and use block grants or per capita caps seems to be on the back burner at the time of this writing, these proposals are likely to resurface. Furthermore, dozens of states are moving forward to restructure their Medicaid programs under the §1115 waiver authority with the aim of reducing enrollment and ultimately reducing spending. There is a considerable degree of uncertainty and speculation surrounding such waivers (including those that have already been approved), due to the lack of information regarding the implementation and enforcement of the new waiver requirements and, in some instances, in‐progress litigation.

While many health centers are taking a “wait and see” approach as a consequence of this uncertainty, some of the health center leaders we interviewed discussed their ongoing or planned efforts to educate staff about new or anticipated requirements and prepare them to best help patients remain covered. Some health centers have also begun to project the budgetary impacts at various levels of Medicaid coverage loss among their patient population. Across the board, interviewees stated that they would benefit from states’ timely and ongoing communication regarding the day‐to‐day implementation of proposed and approved changes to the Medicaid program.

Finally, although a number of our interviewees expressed skepticism about the willingness and capability of state governments to step in and recover the federal revenue losses in the event of block grants or per capita caps, we stress that states should plan for shortfalls by setting aside additional funds to make up for at least some of the difference to support the continued operations of health centers, and ultimately to protect continued health care access for the Medicaid‐dependent population. We further note that these set‐asides should not be limited to a one‐time appropriation but should be authorized and appropriated yearly and preferably for a longer time frame, given the annually compounding impacts of proposals such as block grants and per capita caps.

## Conclusion

Our study contributes to the existing research on Medicaid per capita caps and block grants by focusing on the potential impact they can have on health centers as primary care providers for low‐income populations. Within the confines of our assumptions and what we were able to model, our findings confirmed that both per capita caps and block grants would result in similar, high levels of revenue losses for health centers across the United States, and these billion dollar losses would have serious implications for their ability to operate. Considering these results and the original goal of the Medicaid program, coupled with the literature pointing to an overall positive impact of Medicaid expansions, which has lowered the uninsured rate, provided financial protection, and improved access to needed services, one ultimately has to ask, as some did in our interviewees, “What's the end goal here?” As noted by participants in our interviews, changes such as block grants, per capita caps, and various §1115 waiver requirements may represent the beginning of a shift toward an open‐market, system‐based Medicaid program. While this would achieve the goal of reducing Medicaid's cost burdens to the federal government, health access and outcomes for vulnerable populations would almost certainly be compromised.

Repealing and replacing the ACA and use of block grant funding of the Medicaid program were proposals included in the budget President Donald Trump submitted to Congress in early March 2019, demonstrating that federal Medicaid cutbacks (specifically in the form of block grants) remain a goal of the current administration. In addition, the history of the Medicaid program suggests that the idea of Medicaid as a block grant program is likely to resurface periodically in the future. Our simulation results provide important data that empower states and health center leaders to position themselves in future debates around block grants and per capita caps.

## Supporting information

Online Appendix
