# Supplementary material for: Predicting the Impact of Transforming the Medicaid Program on Health Centers’ Revenues and Capacity to Serve Medically Underserved Communities
Source: Milbank Q. 2019 Oct 16;97(4):1015–61. doi: 10.1111/1468-0009.12426 (PMC6904260; doi:10.1111/1468-0009.12426)
Supplement: Supplementary file 1 — Online Appendix [file MILQ-97-1015-s001.docx]

**Online Appendix**

*Baseline Model*

The baseline model (S0) assumed business as usual, and future projections were based on regression models specified in the article. We used population growth from the Cooper Center projections^50^ as exogenous variables.

*Phase-Out of EFMAP from ACA Model*

In this model (S1), we assumed that the federal government would match funding for the Medicaid expansion population at the same levels used for traditional Medicaid beneficiaries (ie, regular FMAP rates). In this model, phase-out would take effect immediately in 2020. The 2018 FMAP rates were used as the FMAP rates for all subsequent years.

*Block Grant Model*

In the block grant model (S2), we essentially followed the BCRA block grant proposal, except we used 2016 instead of 2018-2019 as the year to calculate baseline Medicaid enrollment and target spending (because the required data for 2018-2019 were not available). We estimated the baseline enrollment by multiplying the number of Medicaid patients in 2014 by the US Census population growth rate from 2014 to 2016. For the 2016 baseline spending target, we started with the average per-patient Medicaid spending in 2014-2015 and adjusted that amount for inflation, using an inflation rate derived from CPI-M data. Then, we multiplied the baseline target spending by the baseline enrollment to calculate the baseline block grant payment. Both expansion and traditional Medicaid populations were included in the block grant in this model. We then inflated this payment using CPI-M projections to the period 2020-2024.

*Per Capita Cap Model*

In the PC cap model (S3), we essentially followed the Graham-Cassidy Amendment (GCHJ) Medicaid flexibility . We used the average Medicaid-per-patient spending in 2014 and 2015, inflated to 2016 levels, as the baseline spending target (ie, per capita cap). CPI-M was used to set the inflation rate for the expansion population and CPI-M + 1 percentage point was used for the traditional population. Both expansion and traditional Medicaid populations were subject to the per capita cap in this model.

GCHJ offered the secretary of HHS some flexibility in adjusting the PC cap. We therefore made the following assumptions regarding how the secretary would make adjustments:

- For states whose spending in the previous year was 25% to 30% higher than the national average, the target spending of the following year would be reduced by 0.5%.
- For states whose spending in the previous year was 31% to 35% higher than the national average, the target spending of the following year would be reduced by 1%.
- For states whose spending in the previous year was 36% to 40% higher than the national average, the target spending of the following year would be reduced by 1.5%.
- For states whose spending in the previous year was ≥41% higher than the national average, the target spending of the following year would be reduced by 2%.

*Medical Inflation Rates*

Medical inflation rates up to 2018 were calculated from CPI-M data obtained from the Bureau of Labor Statistics (BLS). To estimate medical inflation after 2018, we used CBO’s 10-year projection of CPI-U from April 2018 and 2018 CPI-U data as predictors to project CPI-Ms into the future using linear regressions.

**Patient population projection models**

Model for estimating Medicare-insured patient population:

${Patients}_{s, t,Medicare}=\gamma_{o}+ \gamma_{1}P_{s,t,65+}E_{s,t,Adults}+\gamma_{2}P_{s,t,65+}$ [A1]

Model for estimating other publicly-insured patient population:

${Patients}_{s,t,OthPubIns}={\pi_{0}+\pi}_{1}P_{s,t,19-64}$ [A2]

Model for estimating privately-insured patient population:

$${Patients}_{s, t,Private}={\delta_{0}+\delta}_{1}P_{s, t,19-49, Female}E_{s,t,Parents+}$$

$\delta_{2}P_{s,t,19-64}E_{s,t,Adults}+\delta_{3}P_{s,t,1-18}E_{s,t,Child}+\delta_{4}P_{s,t,19-64}HIM$ [A3]

Model for estimating uninsured patient population:

$${Patients}_{s,Uninsured}={\sigma_{0}+\sigma}_{1}P_{s,t,1-18}E_{s,t,Child} +\sigma_{2}P_{s,t,19-49,Female}E_{s,t,Parents}+$$

$\sigma_{3}P_{s,t,19-64}E_{s,t,Adults}+ \sigma_{4}P_{s,t,65+}E_{s,t,Adults}+\sigma_{5}P_{s,t,Adult}HIM$ [A4]

where:

- *P* is the number of persons in each population group
- *E* is eligibility income threshold (as a ratio of FPL) for each population group
- *s* indexes states
- *t* indexes years
- *HIM* is a dummy variable for post-implementation of the Health Insurance Marketplace and individual mandate

**Revenue projection models**

Model for estimating per capita Medicare revenues:

$R_{s,t,Medicare}=\alpha_{0}+\alpha_{1}R_{s,t-1,Medicare}+\alpha_{2}t$ [A5]

Model for estimating per capita revenues from other public insurance programs:

$R_{s,t,OthPubIns}=\beta_{1}P_{adults}$ [A6]

Model for estimating per capita private insurance revenues:

$R_{s,t,Private}=\alpha_{0}+\alpha_{1}R_{s,t-1,Private}+\alpha_{2}t+a_{3}t*HIM$ [A7]

Model for estimating per capita self-pay revenues:

$R_{s,t,Uninsured}=\alpha_{0}+\alpha_{1}R_{s,t-1,Uninsured}+\alpha_{2}t+a_{3}t*HIM$ [A8]

where:

- *R* is per capita revenue from each payer source
- *s* indexes states
- *t* indexes years
- *HIM* is a dummy variable for post-implementation of the Health Insurance Marketplace and individual mandate

**Labor cost projection models**

Model for estimating mental health service costs:

$C_{s,t,Mental}={{(\beta}_{0}+\beta}_{1s}{Mental\_FTE}_{HC,t})*\left( 1+r_{s} \right)^{t}$ [A9]

Model for estimating dental service costs:

$$C_{s,t,Dental}=(\beta_{0}+$$

$\beta_{1,s}{Dentist\_FTE}_{HC,t}+\beta_{2,s}{Hygienist\_FTE}_{HC,t}+{\beta_{3,s}DentalAsst\_FTE}_{HC,t})*\left( 1+r_{s} \right)^{t}$ [A10]

Model for estimating other service costs:

$C_{s,t,OthServ}=(\beta_{0}+\beta_{1,s}{OtherServ_{FTE}}_{HC,t})*\left( 1+r_{s} \right)^{t}$ [A11]

Model for estimating administrative and enabling service costs:

$C_{s,t,Admin}=(\beta_{0}+\beta_{1,s}{Admin\_FTE}_{HC,t}+\beta_{2.s}TotalPatients)*\left( 1+r_{s} \right)^{t}$ [A12]

where:

- C represents service-category costs
- *s* indexes state
- *HC* indexes health center
- *t* indexes year
- *r* represents annual labor cost growth

| **Appendix Table 1a.** Projected Effects of Medicaid Change Scenarios on Health Center Revenues, Baseline to 2024: Expansion States^a^ | | | |
| --- | --- | --- | --- |
| **State** | **Percent Revenue Change** | | |
|  | **Phase-Out EFMAP** | **Block Grant** | **Per Capita Cap** |
| Washington | -43 | -42 | -43 |
| Kentucky | -20 | -29 | -21 |
| Indiana | -19 | -27 | -19 |
| Rhode Island | -18 | -26 | -20 |
| Ohio | -17 | -21 | -17 |
| California | -17 | -23 | -18 |
| New York | -15 | -19 | -15 |
| Pennsylvania | -15 | -23 | -17 |
| Colorado | -14 | -13 | -14 |
| New Mexico | -14 | -24 | -16 |
| Connecticut | -12 | -17 | -12 |
| Arkansas | -11 | -20 | -11 |
| District of Columbia | -9 | -16 | -17 |
| Michigan | -7 | -8 | -7 |
| Delaware | -6 | -7 | -6 |
| Arizona | -6 | -12 | -6 |
| Minnesota | -6 | -9 | -6 |
| Nevada | -6 | -8 | -8 |
| New Jersey | -5 | -3 | -5 |
| North Dakota | -5 | -7 | -5 |
| West Virginia | -3 | 0 | -3 |
| Vermont | -2 | -8 | -2 |
| Massachusetts | -2 | -1 | -2 |
| Oregon | -2 | -10 | -7 |
| Louisiana | -2 | -3 | -2 |
| Hawaii | -1 | -10 | -1 |
| Illinois | -1 | 5 | -1 |
| Maryland | -1 | -1 | -2 |
| Alaska | -1 | -5 | -2 |
| Montana | 0 | -3 | 0 |
| Iowa^b^ | NA | NA | NA |
| New Hampshire^b^ | NA | NA | NA |

Abbreviations: EFMAP, enhanced Federal Medical Assistance percentage; NA, not applicable.

^a^Expansion status as of March 2018.

^b^Data are not available for Iowa and New Hampshire because their Medicaid expansion populations received subsidies for private qualified health plans, which skew revenue estimates.

| **Appendix Table 1b.** Projected Effects of Medicaid Change Scenarios on Health Center Revenues, Baseline to 2024: Non-expansion States^a^ | | | |
| --- | --- | --- | --- |
| **State** | **Percent Revenue Change** | | |
|  | **Phase-Out EFMAP** | **Block Grant** | **Per Capita Cap** |
| Florida | 0 | -4 | -1 |
| South Carolina | 0 | -3 | -1 |
| Maine | 0 | 0 | 0 |
| Texas | 0 | 0 | 0 |
| Wyoming | 0 | 0 | 0 |
| Idaho | 0 | 0 | 0 |
| South Dakota | 0 | 0 | 0 |
| Nebraska | 0 | 0 | 0 |
| Utah | 0 | 1 | 0 |
| Virginia | 0 | 1 | 0 |
| Georgia | 0 | 1 | 0 |
| North Carolina | 0 | 2 | 0 |
| Mississippi | 0 | 3 | 0 |
| Missouri | 0 | 3 | 0 |
| Alabama | 0 | 4 | 0 |
| Oklahoma | 0 | 4 | 0 |
| Wisconsin | 0 | 4 | 0 |
| Kansas | 0 | 4 | 0 |
| Tennessee | 0 | 5 | 0 |

Abbreviation: EFMAP, enhanced Federal Medical Assistance percentage.

^a^Expansion status as of March 2018.

| **Appendix Table 2a.** Projected Changes in Administrative FTEs Required to Maintain Baseline Medical FTEs and Budget Neutrality by 2024: Expansion States^a^ | | | | | | |  |
| --- | --- | --- | --- | --- | --- | --- | --- |
| **State** | **Percent Change in Administrative FTEs** | | | | | | |
|  | **Without Enrollment Cutbacks** | | | **With Enrollment Cutbacks** | | | |
|  | **Phase-Out EFMAP** | **Block Grant** | **Per Capita Cap** | **Phase-Out EFMAP** | **Block Grant** | **Per Capita Cap** | |
| Washington | -163 | -153 | -163 | -50 | -47 | -50 | |
| Kentucky | -62 | -103 | -65 | -14 | -21 | -17 | |
| Indiana | -48 | -77 | -48 | -22 | -34 | -22 | |
| California | -46 | -67 | -49 | -46 | -66 | -49 | |
| Rhode Island | -43 | -70 | -51 | -25 | -38 | -31 | |
| Ohio | -40 | -52 | -40 | -10 | -13 | -10 | |
| Pennsylvania | -39 | -66 | -43 | -22 | -36 | -26 | |
| New Mexico | -38 | -75 | -45 | -23 | -43 | -29 | |
| Colorado | -36 | -32 | -36 | -14 | -13 | -14 | |
| New York | -34 | -44 | -34 | -25 | -32 | -25 | |
| Connecticut | -27 | -41 | -27 | -20 | -30 | -20 | |
| District of Columbia | -27 | -50 | -54 | -22 | -39 | -48 | |
| Arkansas | -21 | -43 | -22 | -4 | -7 | -4 | |
| Nevada | -17 | -25 | -23 | -16 | -24 | -22 | |
| Michigan | -16 | -18 | -16 | -3 | -3 | -3 | |
| Minnesota | -14 | -22 | -14 | -7 | -11 | -7 | |
| Arizona | -12 | -25 | -12 | -8 | -16 | -8 | |
| Delaware | -11 | -12 | -11 | -3 | -4 | -3 | |
| North Dakota | -10 | -15 | -10 | -8 | -12 | -8 | |
| New Jersey | -10 | -6 | -10 | -10 | -7 | -10 | |
| West Virginia | -7 | -1 | -7 | -4 | -1 | -4 | |
| Massachusetts | -6 | -2 | -6 | -5 | -1 | -5 | |
| Vermont | -5 | -21 | -6 | -4 | -15 | -4 | |
| Louisiana | -4 | -6 | -4 | -2 | -3 | -2 | |
| Oregon | -4 | -20 | -14 | -3 | -17 | -13 | |
| Hawaii | -3 | -23 | -3 | -2 | -13 | -2 | |
| Illinois | -2 | 10 | -2 | -1 | 10 | -1 | |
| Maryland | -2 | -3 | -6 | -1 | -2 | -5 | |
| Alaska | -1 | -10 | -3 | -1 | -8 | -3 | |
| Montana | 0 | -8 | 0 | 0 | -3 | 0 | |
| New Hampshire | NA | NA | NA | NA | NA | NA | |
| Iowa | NA | NA | NA | NA | NA | NA | |

Abbreviations: EFMAP, enhanced Federal Medical Assistance percentage; FTE, full-time equivalent; NA, not applicable.

^a^Expansion status as of March 2018.

^b^Data are not available for Iowa and New Hampshire because their Medicaid expansion populations received subsidies for private qualified health plans, which skew revenue estimates.

| **Appendix Table 2b.** Projected Changes in Administrative FTEs Required to Maintain Baseline Medical FTEs and Budget Neutrality by 2024: Nonexpansion States^a^ | | | |
| --- | --- | --- | --- |
|  | **Percent Change in Administrative FTEs** | | |
| **State** | **Phase-Out EFMAP** | **Block Grant** | **Per Capita Cap** |
| Florida | 0 | -9 | -2 |
| South Carolina | 0 | -7 | -3 |
| Maine | 0 | -1 | 0 |
| Texas | 0 | 0 | 0 |
| Wyoming | 0 | 0 | 0 |
| Idaho | 0 | 1 | 0 |
| South Dakota | 0 | 1 | 0 |
| Nebraska | 0 | 1 | -1 |
| Utah | 0 | 2 | 0 |
| Virginia | 0 | 2 | 0 |
| Georgia | 0 | 3 | 0 |
| North Carolina | 0 | 4 | 0 |
| Mississippi | 0 | 5 | 0 |
| Missouri | 0 | 5 | 0 |
| Alabama | 0 | 6 | 0 |
| Oklahoma | 0 | 8 | 0 |
| Kansas | 0 | 9 | 0 |
| Tennessee | 0 | 9 | 0 |
| Wisconsin | 0 | 10 | 0 |

Abbreviations: EFMAP, enhanced Federal Medical Assistance percentage; FTE, full-time equivalent.

^a^Expansion status as of March 2018.
